# Anti‐SARS‐CoV‐2 Spike IgA2 Induces Inflammation by Human Macrophages

**DOI:** 10.1002/eji.70068

**Published:** 2025-10-06

**Authors:** Lynn Mes, Jennifer Veth, Julie Van Coillie, Jim B. D. Keijser, Elise Mantel, Richard van der Mast, Theo Rispens, Gestur Vidarsson, Marjolein van Egmond, Jeroen den Dunnen, Hung‐Jen Chen

**Affiliations:** ^1^ Center for Infection and Molecular Medicine Amsterdam University Medical Center (UMC) University of Amsterdam Amsterdam The Netherlands; ^2^ Amsterdam Institute for Immunology and Infectious Diseases Amsterdam The Netherlands; ^3^ Department of Experimental Immunohematology Sanquin Research Amsterdam The Netherlands; ^4^ Department of Immunopathology and Landsteiner Laboratory Sanquin Research Amsterdam The Netherlands; ^5^ Department of Molecular Cell Biology and Immunology Amsterdam UMC Vrije Universiteit Amsterdam Amsterdam The Netherlands

**Keywords:** COVID‐19, IgA, inflammation, immunometabolism, macrophage

## Abstract

Severe COVID‐19 is an immunological disorder characterized by a hyper‐inflammatory reaction of the immune system. SARS‐CoV‐2 anti‐spike antibodies of the IgG isotype are known to strongly contribute to this hyperinflammation by overactivation of alveolar macrophages. However, while the pathogenic function of IgG has been extensively studied, very little is known about the function of IgA, the most abundant immunoglobulin isotype in the airways. Although IgA is generally considered noninflammatory, in this study, we show that anti‐spike IgA induces pronounced proinflammatory responses. We demonstrate that stimulation of macrophages with anti‐spike IgA immune complexes in combination with a viral stimulus amplifies proinflammatory cytokine production. This IgA‐induced inflammation is particularly driven by IgA2, the IgA subclass that is increased in the plasma of severely ill COVID‐19 patients. We identified that IgA2‐induced inflammation is predominantly dependent on FcαRI‐Syk signaling. Mechanistically, IgA2‐induced inflammation is linked to enhanced glycolysis and altered mitochondrial function, indicating subclass‐specific immunometabolic reprogramming. Taken together, these data indicate a pathogenic role for IgA2 in severe COVID‐19 and highlight its signaling cascades and metabolic pathways as potential druggable targets to counteract hyperinflammation in severe coronavirus infections, such as COVID‐19, SARS, MERS, and potential future outbreaks.

## Graphical abstract

Anti‐SARS‐CoV‐2 spike IgA drives inflammation in human macrophages through FcαRI‐Syk signaling. IgA2 immune complexes amplify cytokine production and alter glycolysis and mitochondrial function more potently than IgA1, indicating subclass‐specific immunometabolic reprogramming. These findings identify IgA2 as a pathogenic factor in severe COVID‐19 and highlight pathways with potential therapeutic relevance.

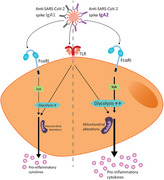

**Abbreviations**
BAL: bronchoalveolar lavage
COVID‐19: coronavirus disease 2019
DC: dendritic cell
FcR: Fc receptor
Ig: immunoglobulin
IL: interleukin
MTT: 3‐[4,5‐dimethylthiazol‐2‐yl]‐2,5 diphenyl tetrazolium bromide
OCR: oxygen consumption rate
PER: proton efflux rate
Poly(I:C): polyinosinic:polycytidylic acid
SARS‐CoV‐2: severe acute respiratory syndrome coronavirus 2
SDH: succinate dehydrogenase
TLR: toll‐like receptor

## 1 Introduction

Severe acute respiratory syndrome coronavirus 2 (SARS‐CoV‐2) infections can lead to coronavirus disease 2019 (COVID‐19), which, in most cases, presents with mild symptoms, such as fever, cough, runny nose, headache, and fatigue [1, 2, 3]. Mild patients usually recover within a few weeks. However, some infected individuals progress to severe COVID‐19. This dramatic worsening of the disease occurs approximately 1.5 weeks after infection, which coincides with seroconversion, and is strongly linked to overactivation of the immune system in response to the virus [3, 4], leading to cytokine storms and tissue damage in the lungs [5, 6]. An important cause of this hyperinflammatory response after seroconversion is immunoglobulins (Ig) directed against the spike protein of SARS‐CoV‐2. First, severely ill COVID‐19 patients have extremely high titers of anti‐spike IgGs compared with patients with mild disease [7, 8]. Second, severely ill patients produce anti‐spike IgG with aberrant glycosylation of the Fc tail, which makes these antibodies extremely proinflammatory, thereby inducing hyperactivation by both alveolar macrophages and platelets through enhanced stimulation of their Fc gamma receptors (FcγRs) [8, 9].

The pathogenic function of IgG has been extensively studied in severe COVID‐19 [8, 10, 11, 12]. Yet, little is known about the function of IgA, the main antibody isotype of the airways. IgA can be detected in early phases of COVID‐19, initiating the first neutralizing response to the virus [13]. In general, IgA is well known for such noninflammatory regulatory functions. While these anti‐inflammatory properties of IgA have been extensively described in the literature, its proinflammatory role has generally been disregarded. IgA immune complexes, which are usually formed upon antigen recognition during infection, can activate human myeloid immune cells, such as neutrophils. Through binding of the IgA Fc receptor, FcαRI, and subsequent signaling cascades, this leads to neutrophil extracellular trap (NET) formation and inflammatory cytokine production [14, 15]. However, in most myeloid immune cell types, such as macrophages or dendritic cells (DCs), simultaneous stimulation of FcαRI and PRRs is required for robust inflammatory effects, such as potent proinflammatory cytokine production [16, 17, 18].

SARS‐CoV‐2‐specific IgA levels are most abundant in the saliva of infected patients [13], but serum IgA levels have been linked to COVID‐19 disease presentation [19, 20]. High systemic IgA levels have been previously described to correlate with disease severity, as ICU‐admitted patients had higher IgA titers in their serum compared with mild cases [13, 19, 20]. Persistently high IgA titers have also been described for fatal COVID‐19 cases [21]. The bronchoalveolar lavage of these patients was dominated by disease‐specific IgA immune complexes over IgG, along with a decreased neutralizing capacity, distinguishing them from survivors [22]. However, the exact role of IgA in severe COVID‐19 and the linked overactivated immune responses remains to be elucidated.

There are two IgA subclasses, IgA1 and IgA2, which have subtle structural differences and, therefore, can have distinct functionalities [23, 24]. We have previously shown that IgA subclasses can play a role in the inflammatory response in myeloid cells, where IgA2 induces higher levels of inflammation in DCs specific for the lower intestine [18]. Moreover, elevated levels of virus‐specific IgA2 have been previously linked to systemic inflammation and fatal outcomes of COVID‐19 patients [25]. Since IgA2 levels are specifically elevated in severe, but not mild, SARS‐CoV‐2 infection, a role as a “mechanistic marker” for severe COVID‐19 was suggested [26].

Here, we set out to investigate the immune responses induced by IgA in macrophages. We show that anti‐spike IgA complexes amplify proinflammatory cytokine production by macrophages upon viral stimulation, with IgA2 inducing more pronounced inflammatory responses than IgA1. Moreover, IgA2‐induced inflammation exhibits a stronger reliance on glycolysis and stimulates mitochondrial dehydrogenase activity, suggesting the involvement of immunometabolic alterations in the underlying regulatory mechanisms.

## 2 Results

### 2.1 IgA Immune Complexes Synergistically Promote Proinflammatory Cytokine Production by Human Alveolar‐Like Macrophages

**FIGURE 1:**
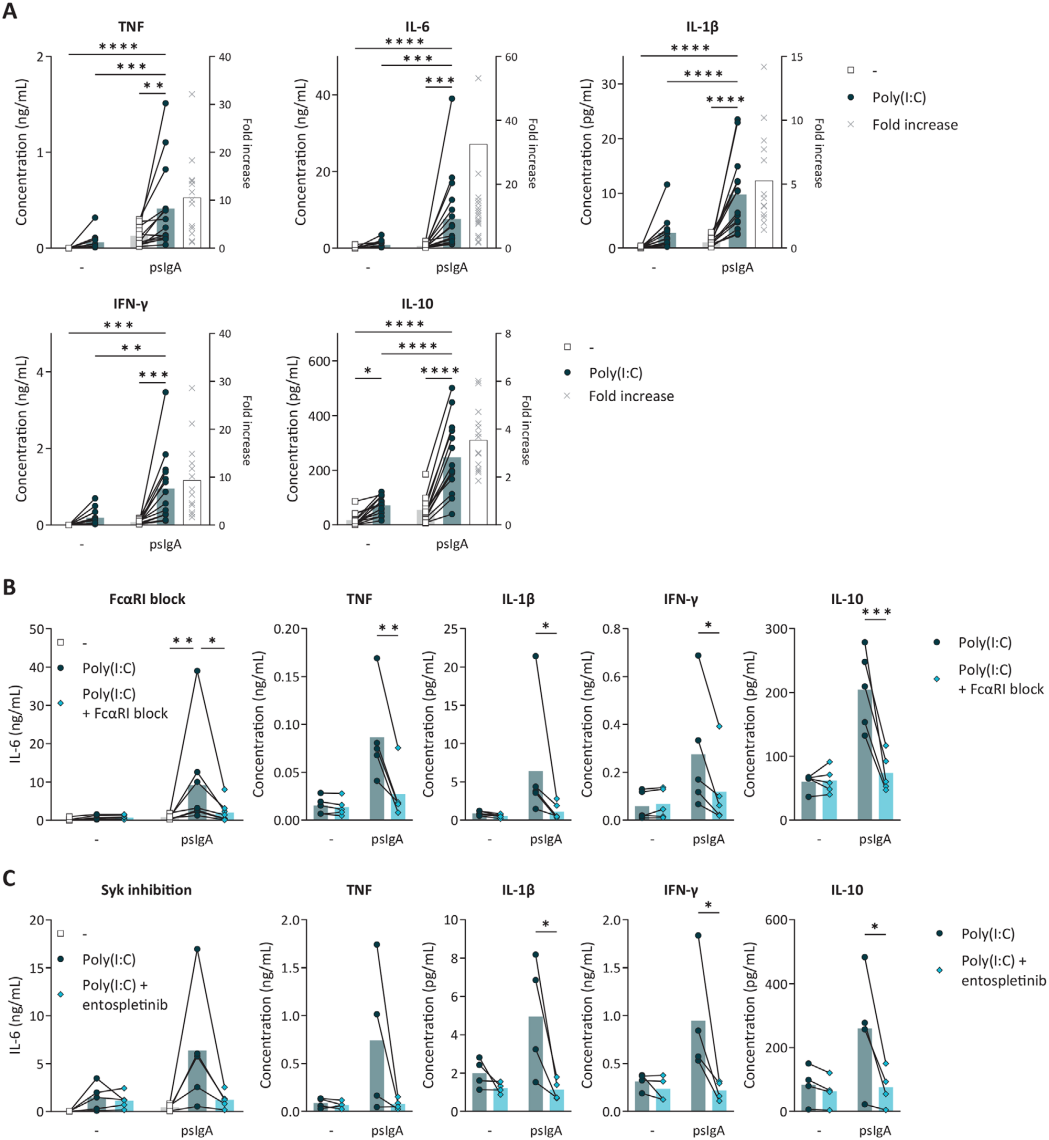
IgA induces proinflammatory cytokine production by alveolar‐like macrophages. Macrophages were stimulated with immune complexes made from pooled serum (ps) IgA and TLR3 agonist Poly(I:C) and analyzed for their cytokine production (**A**). Cytokine concentrations are depicted on the left *y*‐axis, and the fold increase of cytokine production upon double stimulation with psIgA and Poly(I:C) over single stimulation with Poly(I:C) is depicted on the right *y*‐axis (*n* = 14–19). After Preincubation with FcαRI blocking antibodies (**B**) or Syk inhibitor entospletinib (**C**), macrophages were stimulated with psIgA and Poly(I:C) and analyzed for their cytokine production (*n* = 4–8). Symbols represent one individual donor, and bars indicate mean values. *p < 0.05, **p < 0.01, ***p < 0.001, ****p < 0.0001 (two‐way ANOVA).

To assess the inflammatory potential of IgA immune complexes in the context of severe COVID‐19, we used our previously developed *in vitro* human monocyte‐derived macrophage model using IL‐10 activation [8, 27]. This macrophage model resembles alveolar macrophages [28], with increased expression of alveolar macrophage marker MARCO [29, 30] and tissue‐resident macrophage marker CD163 [31] (Figure S1A). Focusing on IgA‐mediated responses, we validated the expression of the main IgA receptor, FcαRI. The macrophages and their monocyte origin showed clear FcαRI expression (Figure S1B). Next, to analyze their responsiveness to IgA, we stimulated these macrophages with immune complexes formed by immobilized pooled serum IgA from healthy donors and toll‐like receptor 3 (TLR3) agonist Polyinosinic:polycytidylic acid (Poly(I:C)). In this model, single stimulation with Poly(I:C) mimics the initial phase of infection, while co‐stimulation with Poly(I:C) and IgA reflects the postseroconversion phase, which occurs around 1.5 weeks postinfection when the disease deteriorates with high levels of inflammation in severely ill patients. Stimulation with Poly(I:C) alone resulted in a modest increase in proinflammatory cytokines, such as TNF, IL‐1β, and IL‐6 (Figure 1A). While stimulation with IgA alone also resulted in mild inflammation, only co‐stimulation with both Poly(I:C) and IgA significantly amplified proinflammatory cytokine production in these macrophages (Figure 1A).

This synergistic effect on IgA‐induced proinflammatory cytokine production was almost completely dependent on FcαRI and the signaling molecule Syk, since blocking with an FcαRI‐neutralizing antibody (Figure 1B) or Syk inhibitor entospletinib (Figure 1C) suppressed all hallmark cytokines assessed, including IL‐6 and TNF. These data indicate that IgA immune complexes, when combined with a viral stimulus, strongly induce inflammation by human alveolar‐like macrophages, which is dependent on the receptor FcαRI and the kinase Syk.

### 2.2 Proinflammatory Cytokine Production Is Mostly Induced by the IgA2 Subclass

**FIGURE 2:**
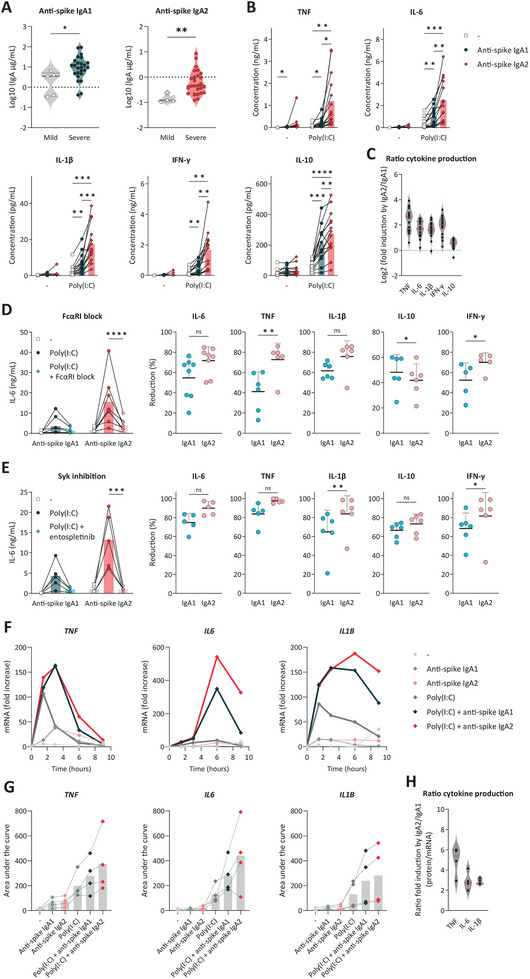
Proinflammatory cytokine production is mostly induced by IgA2. Anti‐spike IgA1 and IgA2 antibody titers in sera of mild and severe COVID‐19 patients were measured by ELISAs (**A**). Each dot represents a single donor (*n* = 5–25); statistical significance was determined using unpaired t‐tests. Upon stimulation with anti‐spike IgA1 or ‐IgA2 and Poly(I:C), alveolar‐like macrophages were analyzed for cytokine production (**B**). Each dot or line represents a donor (*n* = 14), and the average of multiple donors is reflected by bars (two‐way ANOVA). The ratio of protein production by IgA2 over IgA1 is presented in (**C**). The cells were preincubated with FcαRI blocking antibodies (**D**) or Syk inhibitor entospletinib (**E**) to examine the dependency on both the receptor and the signaling molecule. The cytokine concentration of IL‐6 is depicted in the left panel (*n* = 5–8, two‐way ANOVA), and other cytokines in Figure S2. Right panels show the cytokine reduction upon pretreatment as compared with double stimulation with poly(I:C) and IgA (paired t‐tests). Transcriptional regulation was analyzed by qPCR (**F**). mRNA levels were corrected to the housekeeping genes RACK1, HPRT1, and UBB. For every gene of interest, the fold increase compared with unstimulated cells, *T* = 0, is presented for a representative example. The area under the curve of these mRNA expression graphs is depicted in (**G**). Each dot or line represents a donor (*n* = 4), and the average of multiple donors is reflected by bars. For each cytokine, the ratio of protein production over mRNA expression upon IgA2/1 stimulation, in the presence of Poly(I:C), was calculated (**H**). *p < 0.05, **p < 0.01, ***p < 0.001, ****p < 0.0001.

Humans express two distinct types of IgA subclasses, IgA1 and IgA2 [24, 32, 33]. Previous studies have shown that severely ill COVID‐19 patients express higher levels of anti‐spike IgA, particularly IgA2, compared with those with mild symptoms [25, 26]. Similar to these findings, our data also reveal that severe (hospitalized) COVID‐19 patients exhibit increased IgA levels, with a more pronounced increase observed in anti‐spike IgA2, when compared with mild (nonhospitalized) patients in our cohorts (Figure 2A).

To determine whether macrophages have a different sensitivity to IgA subclasses, we stimulated macrophages with monoclonal anti‐spike IgA1 or IgA2 with an identical Fab region and determined proinflammatory cytokine production. While both subclasses induced cytokine production by macrophages upon co‐stimulation with Poly(I:C), IgA2‐driven cytokine production was significantly higher than IgA1 for all measured cytokines, including IL‐1β, IL‐6, IFN‐γ, TNF, and IL‐10 (Figure 2B). Notably, while IgA2 particularly amplified proinflammatory cytokines, it only moderately increased anti‐inflammatory IL‐10 production (Figure 2C). For both subclasses, the inflammatory effects were dependent on FcαRI and Syk (Figure 2D,E). Interestingly, proinflammatory cytokine production induced by IgA2 seemed more dependent on FcαRI, as the reduction of TNF and IFN‐γ was significantly higher than for IgA1, with IL‐6 and IL‐1β showing a similar trend (Figure 2D; Figure S2A). Furthermore, IgA2‐dependent IL‐1β and IFN‐γ production were significantly more reduced upon Syk inhibition than IgA1‐dependent cytokine production, with a comparable trend for TNF and IL‐6 (Figure 2E; Figure S2B).

Further investigating the Syk signaling cascade, we applied multiple inhibitors against subunits of phosphatidylinositol 3 kinase (PI3K), a direct target of Syk. We found that treatment with alpelisib (PI3Kα inhibitor), duvelisib (PI3K‐γ/δ inhibitor), or idelalisib (PI3Kδ inhibitor) all resulted in lower levels of cytokines TNF and IL‐6 in IgA co‐stimulated macrophages, but not in macrophages that were stimulated with Poly(I:C) only (Figure S3A,B). This indicates that IgA‐amplified inflammation is mediated via the FcαRI‐Syk‐PI3K axis.

The mRNA expression of these proinflammatory cytokine genes was found to peak around 3–6 h (Figure 2F). Interestingly, while IgA1 and IgA2 stimulation resulted in pronounced differences in protein production, there were only minor differences in gene expression, with just slightly higher proinflammatory cytokine mRNA levels upon IgA2 stimulation (Figure 2F,G). Hence, the ratio of protein production upon IgA2/IgA1 stimulation was higher than the IgA2/IgA1‐induced mRNA expression ratio for all cytokines tested (Figure 2H). This suggests that the differential proinflammatory effects of IgA subclasses are only partially regulated at the level of gene transcription.

### 2.3 IgA2‐Induced Inflammation Is More Dependent on Glycolysis and Results in Higher Mitochondrial Succinate Dehydrogenase Activity Than IgA1

**FIGURE 3:**
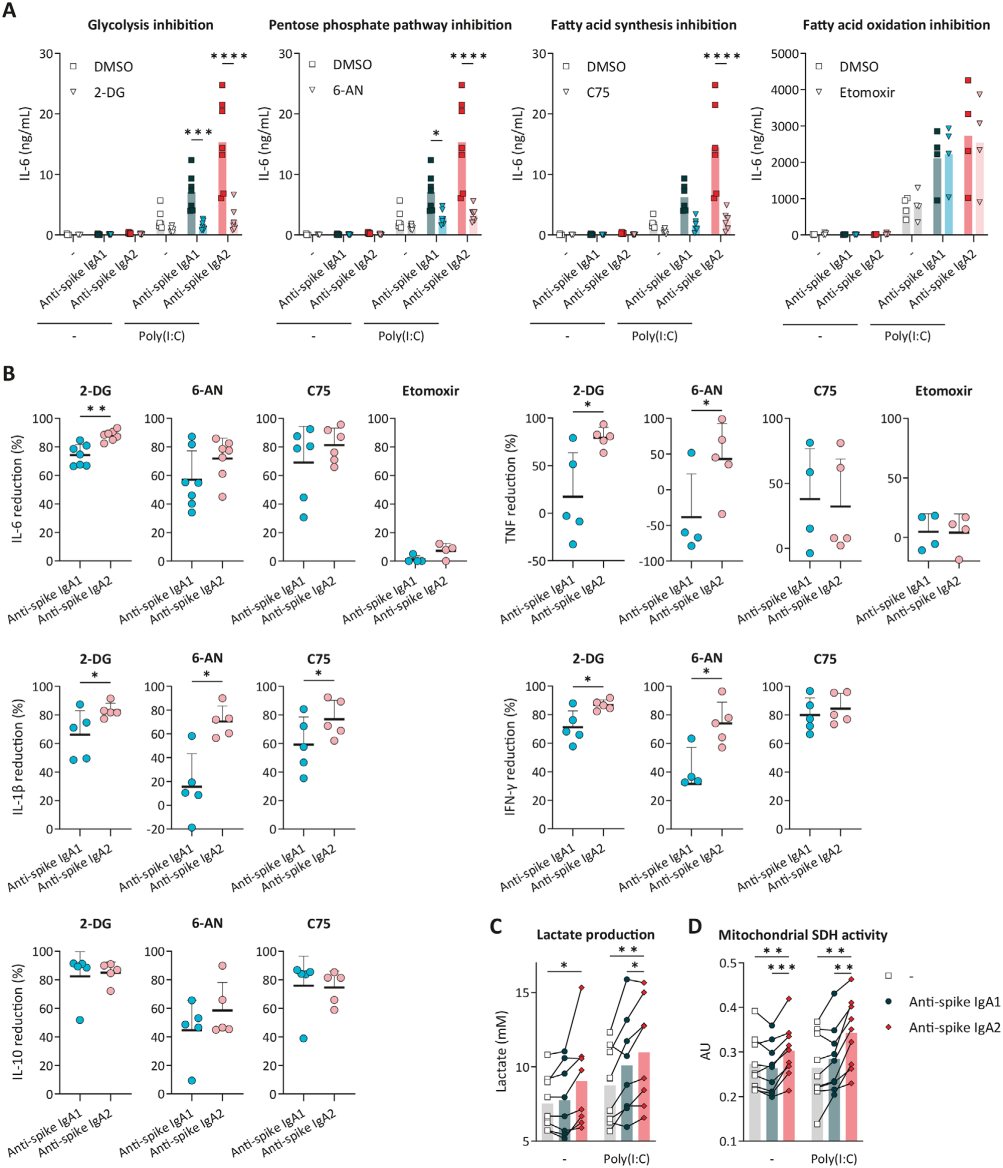
IgA2‐induced inflammation is more dependent on glycolysis and results in higher mitochondrial activity than IgA1. Alveolar‐like macrophages co‐stimulated with Poly(I:C) and anti‐spike IgA were tested for their dependency on glycolysis, the pentose phosphate pathway, and fatty acid metabolism. Cells preincubated with metabolic inhibitors were stimulated and analyzed for their production of IL‐6 (**A**) and other cytokines (Figure S4). Each dot represents a single donor, and bars reflect the mean cytokine production (*n* = 4–7), statistically tested by two‐way ANOVA. The cytokine‐reduction rates compared with co‐stimulation with Poly(I:C) and IgA are shown in (**B**). Data of single donors (dots) with mean + SD are analyzed by paired t‐tests (*n* = 4–7). Samples were also examined for L‐lactate production (**C**) and mitochondrial SDH activity (**D**). Each dot or line represents a donor, and bars represent mean values per condition (*n* = 9–10). Data were tested for statistical significance by two‐way ANOVA, *p < 0.05, **p < 0.01, ***p < 0.001, ****p < 0.0001. SDH, succinate dehydrogenase.

Whereas IgA‐induced inflammation has been linked to cellular metabolic changes [34], it remains unclear whether the two IgA subclasses induce different types of metabolic reprogramming. To study the metabolic dependency of IgA subclass‐dependent inflammation, we incubated macrophages with different metabolic pathway inhibitors before stimulation with anti‐spike IgA and Poly(I:C). In general, IgA‐mediated macrophage cytokine production was critically dependent on glycolysis, the pentose phosphate pathway, and fatty acid synthesis, since inhibition of these metabolic processes with 2‐DG, 6‐AN, and C75, respectively, resulted in reduced IL‐6, IL‐1β, IFN‐γ, and IL‐10 levels (Figure 3A; Figure S4A–D). Inhibition of fatty acid oxidation with etomoxir did not affect cytokine production (Figure 3A; Figure S4A). Interestingly, when comparing the effect of these metabolic inhibitors between the IgA subclasses, only glycolysis inhibitor 2‐DG showed significant differences in the complete proinflammatory cytokine panel (Figure 3B).

To study the association between glycolysis and IgA subclass‐dependent activation in more detail, we measured macrophage lactate production as an indicator of the glycolytic capacity. Upon single stimulation of macrophages with anti‐spike IgA, only IgA2 triggered a significant increase in lactate production, but not IgA1 (Figure 3C). Co‐stimulation with Poly(I:C) resulted in higher lactate production when stimulated with IgA2 as compared with IgA1 (Figure 3C). Further diving into potential downstream mechanisms, we found that glucose transporter 1 (GLUT1), encoded by *SLC2A1*, is particularly induced by IgA2 co‐stimulation (Figure S5A,B). Together, these data indicate that glycolysis is one of the primary metabolic drivers of the differential activation observed between IgA1 and IgA2.

Besides glycolytic reprogramming, we evaluated the cells for their mitochondrial activity using MTT assays, which measure mitochondrial succinate dehydrogenase (SDH) activity. Mitochondrial SDH activity was mostly affected by the IgA2 antibody isotype, since anti‐spike IgA2 stimulation resulted in higher levels of mitochondrial MTT conversion (Figure 3D), highlighting the importance of mitochondrial activity alongside glycolytic reprogramming for IgA2‐specific inflammation.

### 2.4 IgA2 Enhances Glycolysis and Alters Mitochondrial Function in Macrophages

**FIGURE 4:**
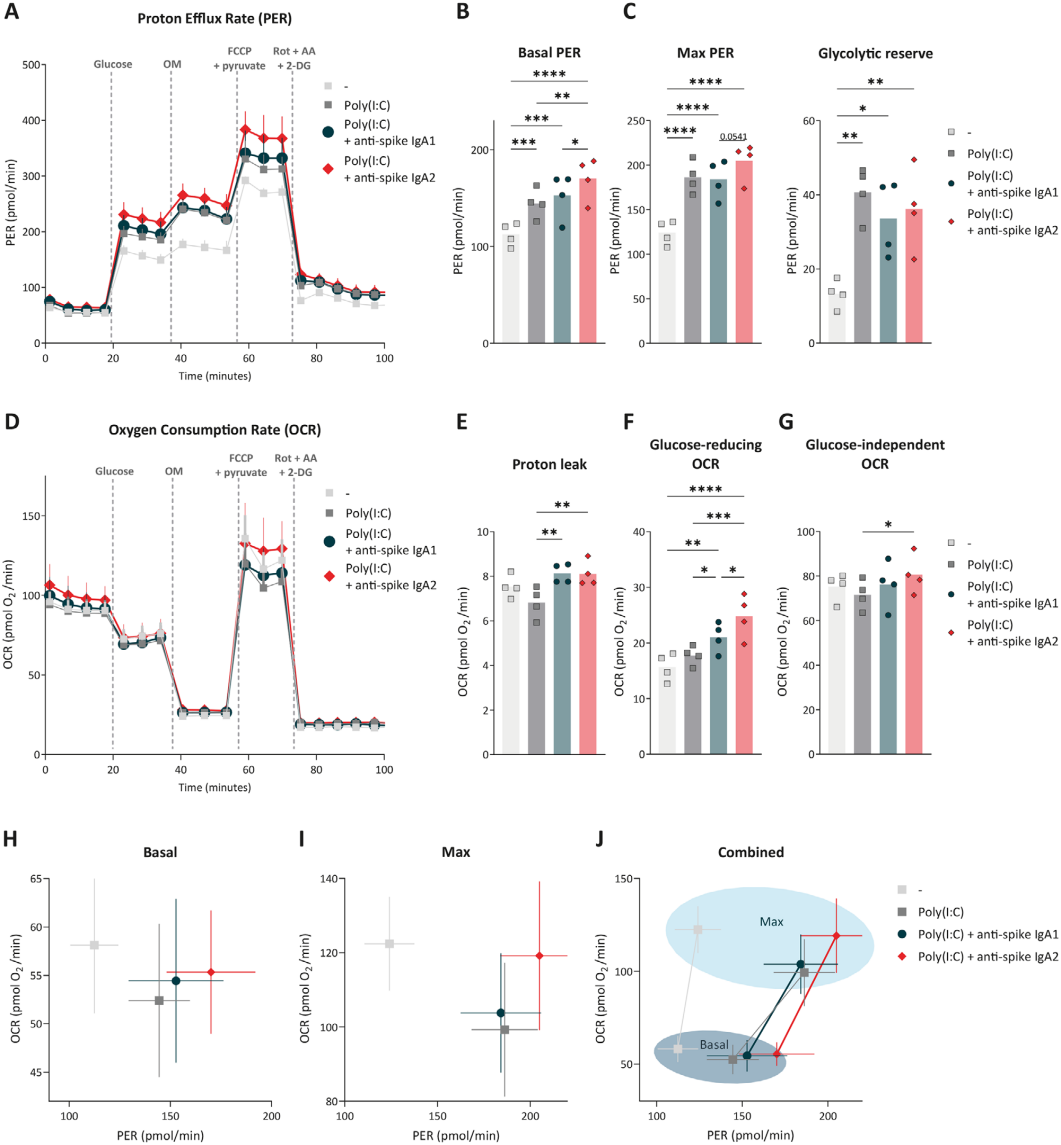
Metabolic reprogramming by IgA2 is characterized by enhanced basal glycolysis and mitochondrial alterations. Extracellular flux assays (Seahorse) were performed on macrophages that were first stimulated with Poly(I:C) and anti‐spike IgA1 and anti‐spike IgA2 for 3 h. During the assay, glucose, oligomycin (OM), FCCP with pyruvate (FCCP + pyr), and rotenone with Antimycin A and 2‐DG (ROT + AA + 2‐DG) were injected at different timepoints. The proton efflux rate (PER) was measured over time (**A**), along with Basal (**B**) and Max PER, and the glycolytic reserve (**C**). The oxygen consumption rate (OCR) (**D**) with calculated proton leak (**E**), glucose‐reducing OCR (**F**), and glucose‐independent OCR (**G**). In panels A and D, each dot represents the mean + SD of four donors. Panels B, C, E–G present the calculated parameters of these donors (dots) and the mean values (bars) per condition, analyzed by one‐way ANOVA. These OCR and PER values are shown for basal (**H**), max (**I**), and combined in (**J**), presented as mean + SD. *p < 0.05, **p < 0.01, ***p < 0.001, ****p < 0.0001.

Given the observed changes in lactate production and mitochondrial SDH activity, we further examined IgA‐induced metabolic reprogramming using Seahorse assays. In line with our lactate measurement, Poly(I:C) stimulation markedly increased glycolysis, as indicated by elevated proton efflux rate (PER) (Figure 4A). Co‐stimulation with IgA2 further enhanced macrophage glycolytic flux beyond Poly(I:C) alone, whereas IgA1 had no additive effect (Figure 4A). Analysis of glycolytic parameters revealed that, compared with macrophages activated by Poly(I:C) alone, IgA2 co‐stimulation primarily augmented basal glycolysis (Figure 4B), without significantly affecting maximal glycolytic capacity or reserve (Figure 4C). Contrarily, basal‐, maximal‐, and reserve oxygen consumption rates (OCR) remained statistically unaffected across stimulation conditions (Figure 4D; Figure S6A–C). However, both IgA1 and IgA2 stimulation induced a significant increase in mitochondrial proton leak (Figure 4E), suggesting altered mitochondrial integrity or efficiency.

To further explore the interplay between glycolysis and aerobic respiration, we followed the OCR changes following glucose injection. The reduction in OCR after glucose addition indicates a shift of energy preference toward glycolytic ATP production, which reflects the dependency of energy production by glycolysis. Both IgA1 and IgA2 enhanced glucose‐induced OCR drop, with IgA2 exerting a stronger effect (Figure 4F). Moreover, compared with the cells stimulated by Poly(I:C) alone, IgA2‐co‐stimulated macrophages show a higher glucose‐independent OCR (defined as the difference between preglucose OCR and postinhibitor OCR) (Figure 4G). Integrating PER and OCR data, we observed that Poly(I:C)‐stimulated macrophages adopt a more glycolysis‐dependent metabolic profile, which is further amplified by IgA2. This shift was evident in the basal energy phenotype (Figure 4H). Under metabolic stress, IgA2‐stimulated cells demonstrated enhanced capacity for both glycolysis and aerobic respiration, as shown by increased maximal PER and OCR values (Figure 4I,J). These results highlight the ability of IgA2 to potentiate macrophage metabolic fitness in inflammatory contexts.

### 2.5 IgA2‐Dependent Inflammation and Metabolic Changes in Different Macrophage Models

To investigate the applicability of our findings across different tissue‐resident macrophage subsets, we generated disease‐associated macrophage models using GM‐CSF [35], with or without IL‐10 activation. The IgA‐induced effects on cytokine production, mitochondrial SDH activity, and lactate production were reproducible amongst distinct macrophage subsets (Figure S7A–C). This indicates that IgA subclass‐dependent hyperinflammation and associated metabolic alterations can be generalized to other macrophage subsets.

## 3 Discussion

Severe COVID‐19 is an immunological disorder characterized by hyperinflammation. While anti‐spike antibodies of the IgG isotype are known to strongly contribute to this inflammation, still little is known about the role of IgA, the predominant isotype in mucosal tissues. Using different *in vitro* macrophage models, we identified that anti‐spike IgA immune complexes strongly promote inflammation by these macrophages. This effect is specifically driven by the IgA2 subclass and is dependent on metabolic processes, particularly glycolysis. These results indicate a proinflammatory role of IgA2, which is likely to further contribute to the hyperinflammation observed in severely ill COVID‐19 patients.

IgA is mostly referred to as the mucosal immunoglobulin responsible for noninflammatory immune regulation at sites of primary pathogen interaction. However, in recent years, a more proinflammatory function of IgA during infection has been described [24, 36]. IgA immune complexes can activate neutrophils and other myeloid immune cells, such as macrophages, through FcαRI, leading to proinflammatory cytokine production. Moreover, IgA can contribute to inflammation in various diseases, including autoimmune disorders, such as rheumatoid arthritis and IgA blistering diseases [24, 37, 38, 39]. In COVID‐19, anti‐spike IgA titers correlate with destructive inflammatory processes seen in severely ill patients, such as CRP, neutrophil activation, and subsequent organ damage and fatal outcome [25]. Temporal dynamics of IgA responses are important for such disease progression. The induction of seral anti‐spike antibodies usually takes place 1–2 weeks after the onset of symptoms, and peaks at 3–4 weeks [13, 40]. Our data shows that IgA stimulation amplifies inflammation by macrophages, therefore indicating that high‐titer anti‐spike IgA may trigger and/or perpetuate a hyperinflammatory status.

In severely ill COVID‐19 patients, the IgA2 isotype is significantly enriched in serum [25, 26]. Since increased epithelial permeability during pulmonary inflammation or acute respiratory distress syndrome (ARDS) allows (nonsecretory) serum IgA to enter the alveolar space [41, 42], anti‐spike IgA2 levels might also be increased in the alveolar space of these severely ill COVID‐19 patients. We found that the production of COVID‐19‐associated proinflammatory cytokines, such as IL‐6 and TNF, was significantly higher upon stimulation with IgA2 compared with IgA1. Interestingly, the subclass‐dependent effect on IL‐10 production was less clear, suggesting IgA2 particularly induces proinflammatory cytokines, but has less effect on anti‐inflammatory IL‐10. These findings are in line with previous studies indicating that IgA2 is the more proinflammatory IgA subclass. Yet, these findings, derived from *in vitro* macrophage models, warrant validation in primary alveolar macrophages to extend their physiological relevance.

The enhanced inflammatory potential of IgA2 has been described for various myeloid cell subsets present in the respiratory tract (e.g., DCs, neutrophils, and macrophages) [18, 24, 36]. However, the distinction between the immune activation features of IgA1 and IgA2 might be tissue‐specific. For example, in monocytes, IgA1 can trigger more potent proinflammatory responses than IgA2 [18]. Moreover, while elevated IgA2 levels have been reported in COVID‐19, it remains unclear whether this response is specific to SARS‐CoV‐2 or also occurs during severe infection with other respiratory viruses. Further research is warranted to determine whether this is a broader feature of (severe) respiratory viral diseases.

Interestingly, both IgA1‐ and IgA2‐induced inflammation is highly dependent on FcαRI, which is known to be expressed on alveolar macrophages from BAL [43, 44, 45] and other tissue resident macrophages [45, 46]. The difference in inflammatory capacity of IgA subclasses could be related to their distinct antibody structures, which may exhibit different binding affinities to FcαRI. For example, the longer hinge region of IgA1, providing greater flexibility and additional glycosylation sites [24, 47, 48], could potentially influence its receptor binding affinity. Furthermore, various splice variants of FcαRI have been described, which may contribute to the observed differences in effector functions [44, 49]. Alternative splicing can result in isoforms with incomplete or absent protein domains, which are involved in extracellular binding or signaling. The affinity of these splice variants for antibody subclasses and possible subsequent functional differences remains to be elucidated.

Both IgA1‐ and IgA2‐induced inflammation are dependent on the signaling molecule Syk. Consequently, the observed differences may be quantitative rather than qualitative, reflecting an increased level of Syk signaling. Theoretically, this could also indicate the involvement of an auxiliary receptor for IgA2 that also signals through Syk. Alternatively, there could be an additional inhibitory receptor for IgA1, which would thereby diminish inflammatory responses upon IgA1 stimulation. There are a few IgA receptors known other than FcαRI, such as pIgR and Fcα/μR, which could potentially play a role in this, and would be interesting to study in future research [50, 51].

In severely ill COVID‐19 patients, anti‐spike IgG shows aberrant Fc tail glycosylation, characterized by low fucosylation and high galactosylation, which strongly promotes inflammation through FcγRs [8, 52]. Glycosylation could also play a role in IgA‐induced inflammation, because it is generally even more heavily glycosylated compared with IgG [47]. The glycosylation profiles of the IgA subclasses are different from each other, with IgA1 expressing N‐glycans that are high in sialic acid and galactose, and IgA2 containing less complex N‐glycans [47]. In addition, the longer hinge region of IgA1 has multiple glycosylation sites expressing highly sialylated O‐glycans. Its shorter, unglycosylated hinge region, stabilizing IgA2 in the mucosa [47], could also cause distinct binding options to glycan receptors present on myeloid immune cells. In addition, severe COVID‐19 patients have specific alterations in their IgA glycosylation profile, with IgA1 O‐glycans having low levels of sialylation and high galactosylation, while IgA2 presents with higher sialylation at certain N‐glycosylation sites [53]. Such disease‐specific IgA subclass differences are interesting to include in future studies to increase the understanding of their contribution to antibody‐induced inflammation.

Although we observed pronounced IgA subclass differences in macrophage inflammatory cytokine protein production, differences at the transcriptional level between IgA1 and IgA2 were limited, suggesting that other regulatory mechanisms underlie the distinct activation capacities. Immunometabolic alterations play a significant role in regulating macrophage phenotypes, as the metabolic processes underlie their pro‐ and anti‐inflammatory capacity [54]. Alveolar macrophages also undergo metabolic reprogramming during severe SARS‐CoV‐2 infection, as overactivated immune responses and disease progression are associated with distinct glucose metabolism [55, 56, 57]. Previously, we have shown that FcγR signaling triggered by IgG immune complexes enhances the glycolytic capacity of macrophages [54]. In this study, we found that IgA‐induced inflammation by macrophages is dependent on at least three metabolic pathways: glycolysis, pentose phosphate pathway, and fatty acid synthesis. Similar to cytokine production, the highly glycolytic state was further induced by TLR co‐stimulation. This finding aligns with the FcαRI‐induced glycolytic reprogramming in intestinal CD103^+^ DCs [34], indicating similar metabolic processes in innate immune cells in both the airways and the intestine.

Since IgG subclasses have been previously linked to differential metabolic reprogramming in macrophages [58], the different anti‐spike IgA subclasses were analyzed for their metabolic reprogramming capacity. IgA2‐induced inflammation seems to be more supported by glycolytic reprogramming than IgA1, as shown by greater reduction of IL‐6, TNF, IL‐1β, and IFN‐γ, but not the anti‐inflammatory cytokine IL‐10, upon 2‐DG treatment, along with increased lactate production. This could be a result of higher energy reliance for a stronger inflammatory status, or due to differential signaling in IgA isotype‐mediated metabolic reprogramming. Notably, GLUT1 (*SLC2A1*) transcription is controlled by Syk [59], further strengthening the role of the FcαRI‐Syk axis and metabolic interplay in regulating macrophage immune responses. Without co‐stimulant Poly(I:C), lactate production is only triggered by IgA2, further demonstrating the subclass‐dependent metabolic shift and aligning with our data suggesting increased glycolytic activity of IgA2‐stimulated cells. In addition, our seahorse assay also reveals IgA2‐enhanced glycolytic capacity in macrophages, further supporting the involvement of glucose metabolism in IgA2‐dependent inflammation. The pronounced decrease in OCR after glucose injection, especially in IgA2‐stimulated macrophages, indicates a metabolic shift toward increased preference for glycolytic ATP production and reduced reliance on oxidative phosphorylation, a hallmark of proinflammatory macrophages. Interestingly, the decreased glucose‐independent OCR in IgA2‐Poly(I:C) co‐stimulated macrophages suggests that these macrophages rely more on energy sources other than glucose for their aerobic respiration. Therefore, these metabolic pathways may form interesting targets for future research and therapeutic intervention.

In addition to glycolysis, alterations in mitochondrial function have been shown to regulate the phenotype of macrophages [54, 60]. Our data indicate that IgA promotes mitochondrial SDH‐mediated MTT conversion in macrophages, specifically upon stimulation with IgA2 immune complexes. This indicates an increase in SDH activity in IgA2‐activated macrophages, in line with a previous publication showing SDH to be critical for the inflammatory response in macrophages [61, 62]. SDH activity is associated with inflammation via increased IL‐1β and decreased IL‐1RA and IL‐10 upon TLR stimulation. However, we found that IgA2 stimulation without Poly(I:C) already resulted in higher mitochondrial activity, suggesting a potential role for specific antibody subclass immune complexes in mitochondrial immune regulation. Targeting succinate oxidation could be a promising treatment strategy in viral infections, for example, via the competitive SDH inhibitor dimethyl malonate (DMM), which has been previously described to efficiently counteract inflammation *in vivo* [62].

In conclusion, our data indicate that anti‐spike IgA2 immune complexes trigger inflammatory activation in macrophages, particularly in the context of severe COVID‐19. The accompanying metabolic alterations highlight a differential role for IgA subclasses in macrophage metabolic reprogramming during antibody‐induced inflammation. These findings may be used for the development of therapeutic strategies for severe COVID‐19 and potentially other IgA2‐associated diseases.

## 4 Methods

### 4.1 COVID‐19 Patient Serum Sample Antibody Titer Determinations

Severe COVID‐19 patient serum samples were provided by the Amsterdam UMC COVID‐19 Biobank, including deferred consent and ethical approval. Sera from mild and severe COVID‐19 patients (Table 1) were analyzed for its immunoglobulin concentration by ELISA. ELISAs were performed according to the protocol described previously [63]. Briefly, samples were incubated for 1 h at room temperature in plates coated with SARS‐CoV‐2 spike protein produced as described before [64]. Anti‐spike IgA1 and IgA2 were detected using clones MH141‐1 and A9604D2 (Southern Biotech) detection antibodies, respectively (Table 3). As calibrator for the respective assays, a COVA1‐18 anti‐RBD clone [64] was engineered with a human IgA1 and IgA2 heavy chain, analogously as described previously for IgG1 and IgG3 [65].

**TABLE 1:** COVID‐19 patient characteristics.

#	Disease severity	Sex	Age	Comorbidities	Outcome	Hospitalization (days)	Sampling (days)
01	Mild	F		N/A	Recovered	N/A	21
02	Mild	F		N/A	Recovered	N/A	<25
03	Mild	F	52	N/A	Recovered	N/A	21
04	Mild	M	31	N/A	Recovered	N/A	5
05	Mild	M	35	N/A	Recovered	N/A	<7
06	Severe	M	63	D, H, P	Recovered	10	25
07	Severe	M	59	A, D, H, O	Recovered	14	21
08	Severe	M	70	CHD	Deceased	15	Unknown
09	Severe	M	66	CCD, O	Recovered	16	23
10	Severe	F	51	O, P	Recovered	15	22
11	Severe	F	71	B	Deceased	14	19
12	Severe	M	61	H, P	Recovered	12	20
13	Severe	F	55	O, P	Recovered	8	15
14	Severe	F	52	H, O	Recovered	6	8
15	Severe	F	46	P	Deceased	6	13
16	Severe	M	63	P	Deceased	4	35
17	Severe	F	50	H, O	Recovered	12	26
18	Severe	M	67	AID, B, P	Recovered	10	Unknown
20	Severe	M	65	CCD, D, H	Recovered	9	19
21	Severe	M	54	CCD	Recovered	24	Unknown
22	Severe	M	59	A, CCD, H	Recovered	25	39
23	Severe	M	74	CPD, H, P	Deceased	30	40
24	Severe	M	61	AID, D, I, O, P	Recovered	29	46
25	Severe	M	60	B, CPD, D, I, O, P	Deceased	22	26
26	Severe	M	72	D, H, P, R	Recovered	24	31
27	Severe	F	64	O, P	Recovered	4	7
28	Severe	M	61	H, O, P	Recovered	5	12
29	Severe	M	59	D, P	Recovered	1	5
30	Severe	M	78	CKD, P	Recovered	Unknown	15

*Note*: Mild patients were individuals who tested positive for COVID‐19, without hospitalization. Severe cases were all admitted to the hospital. In the column “Hospitalization”, the number of days between the onset of symptoms and admission to the hospital is depicted. The time in days between the onset of the disease and sample collection can be found under “sampling”.

Abbreviations: F – female, M – male, A – asthma, AID – autoimmune disorder, B – bacteremia, CCD – chronic cardiac disease, CHD – chronic hematologic disease, CKD – chronic kidney disease, CPD – chronic pulmonary disease (not asthma), D – diabetes, H – hypertension, I – immune supp, O – obesity, P – pneumonia, R – rheumatoid arthritis.

### 4.2 Cell Culture

Monocytes were isolated from Buffy coats of anonymous healthy donors who signed informed consent (Sanquin Blood Supplies). PBMCs were isolated using Lymphoprep (Stemcell Technologies), followed by Magnetic cell separation (MACS) with CD14 MicroBeads (Miltenyi) and LS MACS Columns (Miltenyi) to isolate the monocytes. Monocytes were cultured in six‐well plates at a concentration of 4.5 million cells/well. Cells were cultured for 6 days in Iscove's Modified Dulbecco's Medium (IMDM) (Gibco), supplemented with 5% fetal calf serum (FCS) (Carpicorn), 2 mM L‐glutamine (Gibco), 100 U/mL penicillin and 100 µg/mL streptomycin (1% P/S, Sigma Aldrich), and 50 ng/mL recombinant human M‐CSF (Miltenyi) or 20 ng/mL GM‐CSF (Miltenyi). Complete culture medium was refreshed on day 3. After 6 days, the cells were cultured for 24 h in the presence of 50 ng/mL human recombinant IL‐10 (R&D). To harvest the macrophages at day 7, cells were incubated for 20 min in TrypLE select (Gibco), washed with PBS, and resuspended in IMDM with 5% FCS.

### 4.3 Cell Stimulation

Nunc MaxiSorp high protein‐binding capacity 96‐well plates (ThermoFisher Scientific) were coated with 4 µg/mL IgA antibodies. For stimulation, pooled serum (ps) IgA (MP Biomedicals), or monoclonal anti‐spike IgA1 or anti‐spike IgA2 (Sanquin) was used. Anti‐spike IgA1 and IgA2 were produced according to identical procedures as described previously [64, 66]. After overnight incubation, plates were blocked with PBS 10% FCS for 1 h at 37°C.

Cells were stimulated in a concentration of 50,000 cells/well together with 10 µg/ml Polyinosinic:polycytidylic acid (Poly(I:C)) (Sigma Aldrich). In case of FcαRI block, kinase‐, or metabolic inhibition, cells were incubated with the blocking antibodies or small molecule inhibitors for 1 h at 37°C before stimulation. Cells were preincubated with 20 µg/mL anti‐CD89 (MIP8a; LifeSpan BioSciences), 1 µM entospletinib (Selleck Chemicals), 25 µM alpelisib (Selleck Chemicals), 5 µM duvelisib (MedChemExpress), or 50 µM idelalisib (MedChemExpress). All inhibitors were used in optimized concentrations as described previously [67]. For analysis of metabolic pathways, cells were preincubated with 10 mM 2‐DG (MedChemExpress), 0.1 mM 6‐AN (MedChemExpress), 10 mM C75 (MedChemExpress), or 5 µM Etomoxir (Sigma Aldrich). Supernatant was collected after 24 h stimulation and analyzed for cytokine and lactate production.

### 4.4 Cytokine Production

After 24 h of stimulation, cytokine concentrations in the supernatant were measured using an ELISA or Meso Scale Discovery (MSD) multiplex assay. To measure IL‐6 concentrations, an ELISA kit from R&D was used according to the manufacturer's protocol with standards ranging from 2.9 to 3000 pg/mL in high‐affinity plates (Nunc Maxisorp, ThermoFisher Scientific). A Biotek plate reader was used to measure signal at 450 nm, and samples were analyzed and corrected for 540 nm background signals using Gen5 software.

To measure a cytokine panel containing IL‐1β, IL‐6, IL‐10, TNF, and IFN‐γ, a multiplexing human cytokine assay kit V‐PLEX (MSD) was used according to the manufacturer's protocol as described previously [68]. In short, linkers and biotinylated capture antibodies were coated on the plate, and standards for the proinflammatory panel were reconstituted in assay diluents. The MESO QuickPlex SQ 120 plate reader (MSD) and Discovery Workbench Software (v4.0, MSD) were used to measure and analyze the electrochemiluminescence signal. A four‐parameter logistic fitting model was used to calculate the sample concentrations.

### 4.5 Lactate Production

To measure lactate production upon stimulation of macrophages, a L‐lactate assay was performed as previously described [69]. Supernatant was harvested after 24 h of stimulation. Samples were deproteinized using 3% metaphosphoric acid (Sigma). Lactate concentrations were determined using 5 mg/mL L‐lactate dehydrogenase (Roche) in 0.5 M glycine‐0.4 M hydrazine buffer. Fluorescence was measured at λex/λem = 340‐10/450‐10 nm in a fluorescence plate reader (Biotek) and corrected with premeasurement values and a L‐lactic acid standard (Sigma).

### 4.6 Mitochondrial Succinate Dehydrogenase Activity

Mitochondrial succinate dehydrogenase (SDH) activity of the cells was analyzed by an MTT assay. After 24 h of stimulation, supernatant was removed and 1 mg/mL MTT (3‐[4,5‐dimethylthiazol‐2‐yl]‐2,5‐diphenyl tetrazolium bromide in plain IMDM culture medium) was added to the cells. Cells were incubated for 2–3 h at 37°C with 5% CO_2_, after which MTT solution was removed and lysis buffer (4 mM HCl, 0.1% Tween‐20 in isopropanol) was added for 20 min, shaking. A Biotek plate reader was used to measure the plates at 590 nm; values were corrected for background signals at 690 nm.

### 4.7 Real‐Time Extracellular Flux Analysis

Oxygen consumption rate (OCR) and proton efflux rate (PER) measurements were performed using a Seahorse XFe96 Analyzer (Agilent) as previously described [70]. In short, macrophages (50,000 cells/well) were plated with Poly(I:C) in IgA‐coated XF96 cell plates. After 3 h, the culture media were replaced with XF DMEM assay medium (pH 7.4, Agilent) supplemented with 2 mM glutamine and incubated for 30 min at 37°C without CO_2_. The XF assay protocol involved sequential injections to achieve final concentrations of 10 mM glucose, 1.5 µM oligomycin A, 1.5 µM FCCP, 1 mM pyruvate, 2.5 µM antimycin A, 1.25 µM rotenone, and 10 mM 2‐DG. A total of 19 measurement cycles were performed, each consisting of 2 min mixing and 3 min measuring.

### 4.8 mRNA Expression

Quantitative real‐time PCR was performed to analyze mRNA expression levels. Cells were stimulated and lysed at different time points (0, 1.5, 3, 6, and 9 h). RNA isolation and cDNA synthesis were performed according to manuals using a RNeasy Mini kit (Qiagen) and a high‐capacity cDNA Reverse Transcription Kit (Applied Biosystems, ThermoFisher Scientific), respectively. This was followed by qPCR using a SensiFAST SYBR No‐ROX Kit (Bioline, Meridian Bioscience) and self‐designed primer pairs (Table 2) (Sigma Aldrich). Samples were measured with a LightCycler 480 (Roche).

**TABLE 2:** Sequences of primer pairs used for quantitative real‐time PCR.

Target	Forward sequence	Reverse sequence
*HPRT1*	GACCAGTCAACAGGGGACAT	AACACTTCGTGGGGTCCTTTTC
*IL1B*	AAGATGCTGGTTCCCTGC	GTTCAGTGATCGTACAGGTGC
*IL6*	CCTGAACCTTCCAAAGATGGC	TTCACCAGGCAAGTCTCCTCA
*RACK1*	GAGTGTGGCCTTCTCCTCTG	GCTTGCAGTTAGCCAGGTTC
*SLC2A1*	TCTGGCATCAACGCTGTCTTC	CGATACCGGAGCCAATGGT
*TNF*	GGCGTGGAGCTGAGAGAT	TGGTAGGAGACGGCGATG
*UBB*	GGTCCTGCGTCTGAGAGGT	GCCTTCACATTTTCGATGGTGT

**TABLE 3:** Methods key reagents.

Reagent	Source	Identifier
Antibodies		
Anti‐CD89, MIP8α	LifeSpan BioSciences	LS‐C188187
Mouse‐anti‐human IgA1 clone MH141‐1	Sanquin	In‐house production
Mouse‐anti‐human IgA2 clone A9604D2	Southern Biotech	Cat. 9140‐05
Purified human pooled serum IgA (psIgA)	MP Biomedicals	SKU: 0855906
Chemicals, peptides, and recombinant proteins		
2‐Deoxy‐D‐glucose	MedChemExpress	HY‐13966
3‐[4,5‐Dimethylthiazol‐2‐yl]‐2,5 diphenyl tetrazolium bromide (MTT)	Sigma‐Aldrich	475989
6‐Aminonicotinamide	MedChemExpress	HY‐W010342
Alpelisib	Selleck Chemicals	S2814
C75	MedChemExpress	HY‐12364
Duvelisib	MedChemExpress	HY‐17044
Entospletinib (GS‐9973)	Selleck Chemicals	Cat. S7523
Etomoxir	Sigma‐Aldrich	236020
Human GM‐CSF	Miltenyi Biotec	Cat. 130‐093‐864
Human M‐CSF	Miltenyi Biotec	Cat. 130‐096‐491
Idelalisib	MedChemExpress	HY‐13026
L‐lactate	Sigma‐Aldrich	L7022‐5G
Polyinosinic:polycytidylic acid (Poly(I:C))	Sigma‐Aldrich	Cat. P1530
Recombinant human IL‐10 protein	R&D Systems	Cat. 217‐IL‐025/CF
SARS‐CoV‐2 (Wuhan‐Hu‐1) spike	Sanquin	https://doi.org/10.1126/science.abc5902
Critical commercial assays		
CD14 MicroBeads, human	Miltenyi Biotec	Cat. 130‐050‐201
ELISA MAX Standard Set Human IL‐6	BioLegend	Cat. 430501
High‐capacity cDNA reverse transcription kit	ThermoFisher Scientific	4368814
Multiplexing human cytokine assay kit U‐PLEX	Meso scale discovery, inc.	K15049D, K15047D
RNeasy mini kit	Qiagen	Cat. 74104
SensiFAST SYBR No‐ROX Kit	Bioline	BIO‐98005‐BL
Software		
Discovery Workbench Software version 4.0	Meso scale discovery, inc.	www.mesoscale.com
GraphPad Prism version 10.2.0	GraphPad Software	www.graphpad.com

### 4.9 Statistical Analysis

Statistical analysis was performed with GraphPad Prism software (GraphPad Software, v10.2.0). Cytokine production was analyzed for statistical significance using two‐way ANOVA tests. Anti‐spike IgA1 and IgA2 titers in patient serum were compared by unpaired *t*‐tests. Paired *t*‐tests were applied to cytokine reduction data upon FcαRI blocking, kinase‐, and metabolic inhibition. mRNA area under the curve were analyzed by one‐way ANOVA tests. *p*‐values <0.05 were considered significant and indicated as *, **, ***, or **** for *p*‐values <0.05, <0.01, <0.001, and <0.0001, respectively.

## Author Contributions

**Lynn Mes**: formal analysis, investigation, methodology, visualization, writing‐original draft, writing—review, and editing. **Jennifer Veth**: formal analysis, investigation. **Julie Van Coillie**: formal analysis, investigation, methodology, writing—review and editing. **Jim B.D. Keijser**: formal analysis, investigation, methodology, writing—review and editing. **Elise Mantel**: investigation. **Richard van der Mast**: investigation. **Theo Rispens**: writing—review and editing. **Gestur Vidarsson**: writing—review and editing. **Marjolein van Egmond**: Conceptualization, funding acquisition, supervision, writing—review and editing. **Jeroen den Dunnen**: Conceptualization, supervision, writing—original draft, writing—review and editing. **Hung‐Jen Chen**: Conceptualization, funding acquisition, formal analysis, investigation, methodology, supervision, writing—original draft, writing—review and editing.

## Conflicts of Interest

The authors declare no conflicts of interest.

## Peer Review

The peer review history for this article is available at https://publons.com/publon/10.1002/eji.70068.

## Ethics Statement

All human biological samples were sourced ethically, and their research use was in accordance with the terms of the informed consents under an IRB/EC‐approved protocol. Human serum samples provided by the Amsterdam UMC COVID‐19 Biobank and buffy coats provided by Sanquin were obtained, including deferred and informed consent, respectively. No potentially identifiable human images or data are included in this study. No animal experiments are involved in this manuscript.

## Supporting information




**Supporting File**: eji70068‐sup‐0001‐SuppMat.pdf.

## Data Availability

The data that support the findings of this study are available from the corresponding author upon reasonable request.
