# Supplementary material for: Anti‐SARS‐CoV‐2 Spike IgA2 Induces Inflammation by Human Macrophages
Source: Eur J Immunol. 2025 Oct 6;55(10):e70068. doi: 10.1002/eji.70068 (PMC12501405; doi:10.1002/eji.70068)
Supplement: Supplementary file 1 — Supporting File: eji70068‐sup‐0001‐SuppMat.pdf. [file EJI-55-e70068-s001.pdf]

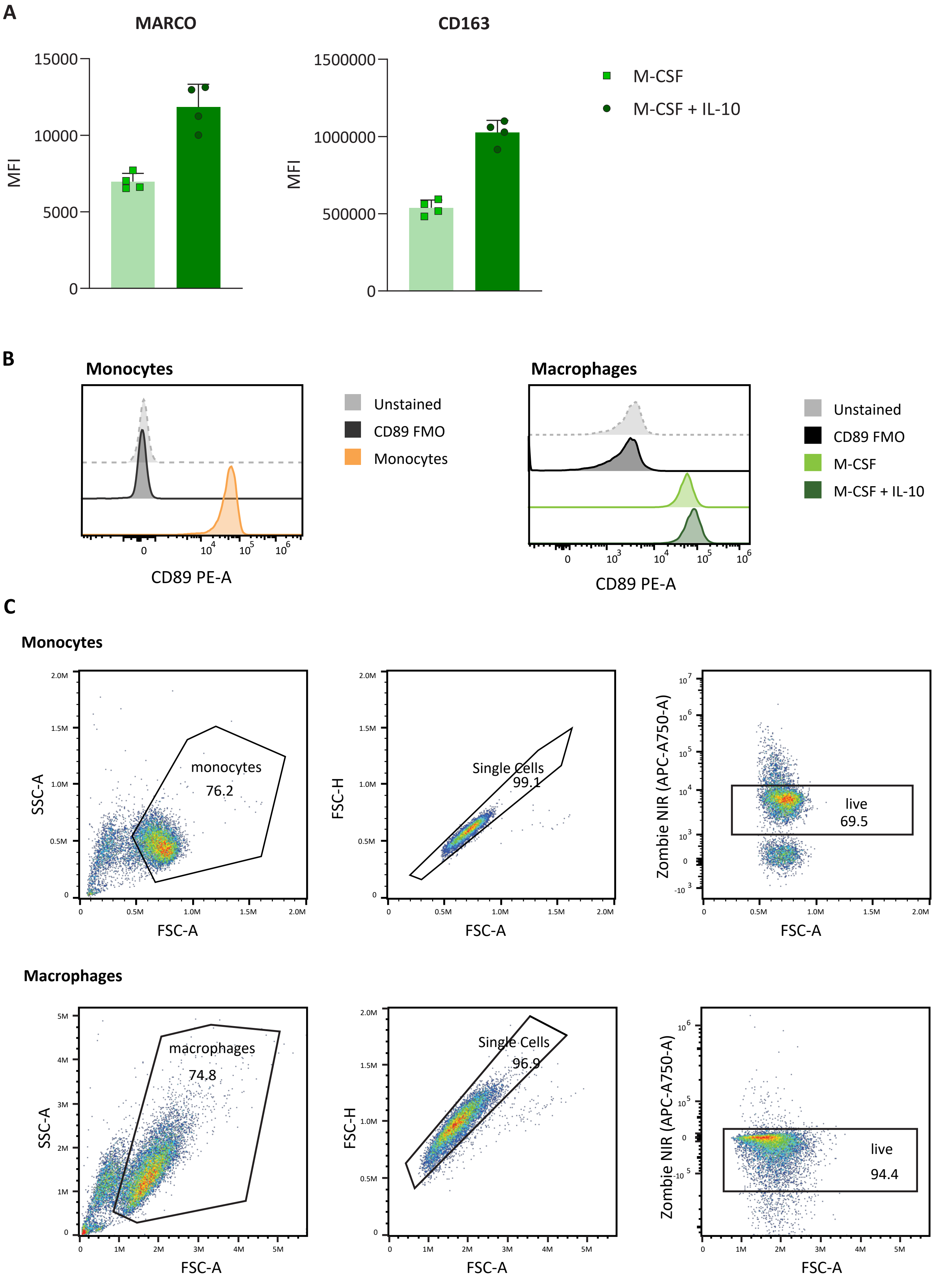

Figure S1. Validation of macrophage phenotypes and FcαRI expression. Macrophages that were differentiated in the presence of M-CSF and stimulated with and without IL-10 were analyzed for the expression of surface markers MARCO and CD163 by FACS (A). The mean fluorescence intensity (MFI) is presented as mean+SD (n=4). The surface expression of FcαRI (CD89) by monocytes (left), and macrophages with or without IL-10 stimulation (right) was also determined by FACS and depicted as representative example (B). The gating strategy of a representative example with population percentages for monocytes (top) and macrophages (bottom) is shown in (C). FMO – fluorescence minus one.

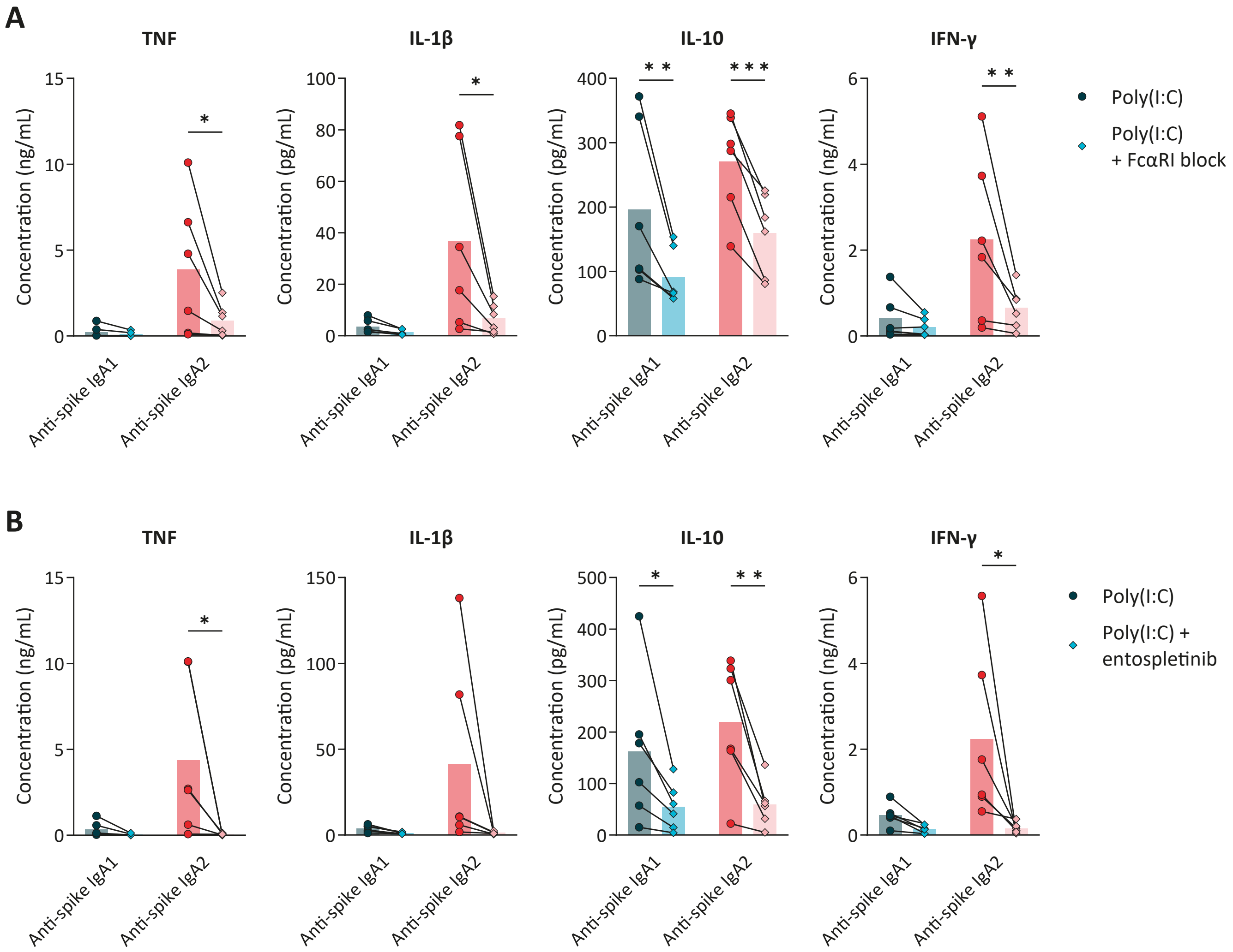

Figure S2. IgA-induced cytokine production is dependent on FcαRI and Syk signaling. Macrophages were pre-incubated with FcαRI blocking antibodies (A) or Syk inhibitor entospletinib (B). The concentration of cytokines beyond IL-6 (Fig. 2) was determined by ELISA (dots and lines represent single donors (n=6), bars represent mean values) and statistically analyzed with two-way ANOVA, $^{*}P<0.05$, $^{**}P<0.01$, $^{***}P<0.001$.

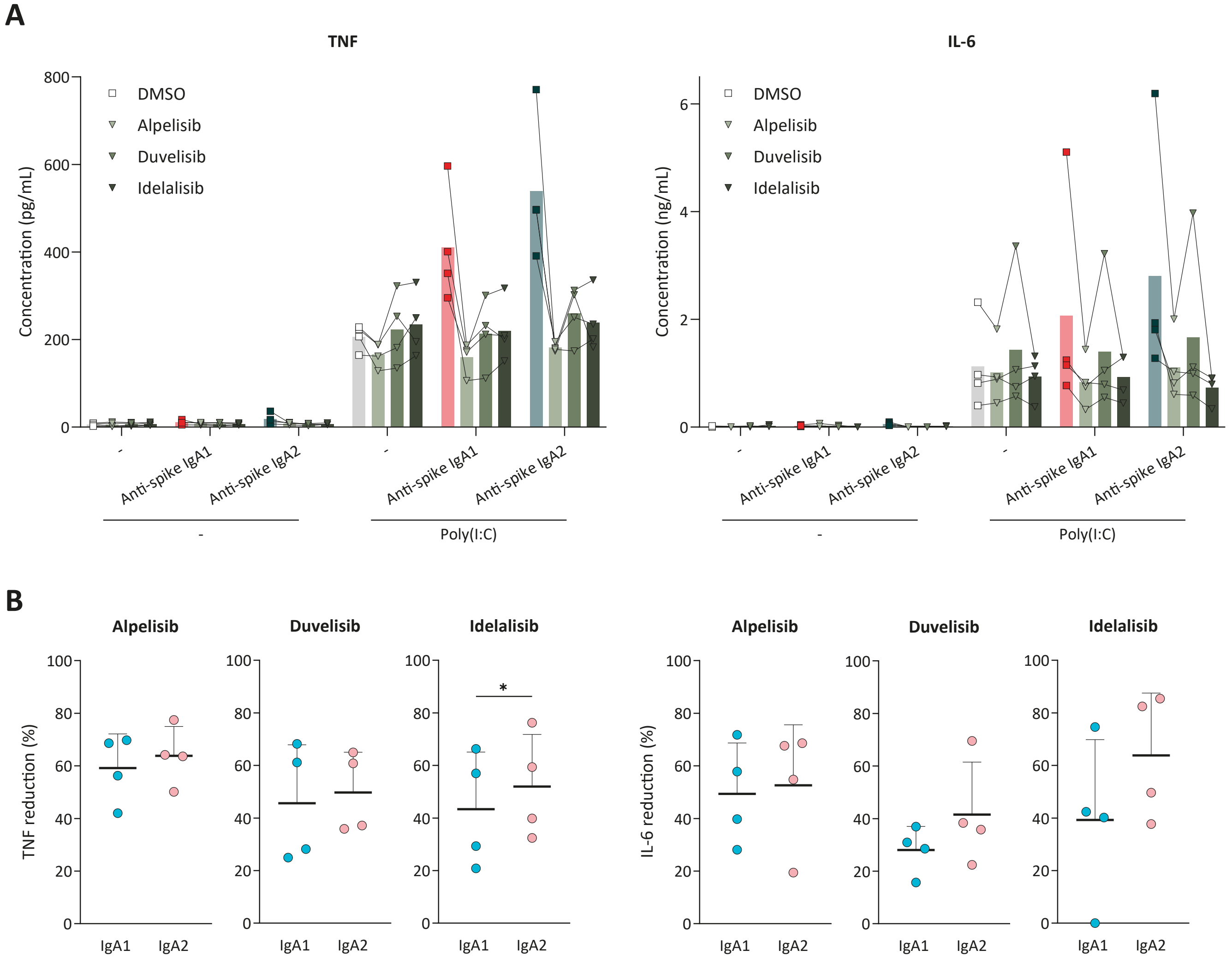

Figure S3. IgA-induced cytokine production and metabolic activity is dependent on Syk signaling through PI3K. Macrophages were incubated with different inhibitors targeting downstream Syk signaling molecule PI3K and stimulated with Poly(I:C) and anti-spike IgA1 or -IgA2. Cytokine production was analyzed by ELISA. TNF and IL-6 concentrations (A) are shown for different donors (n=4, each dot representing a single donor with bars representing mean values). The reduction of cytokine production upon pre-incubation with inhibitors is shown in (B).

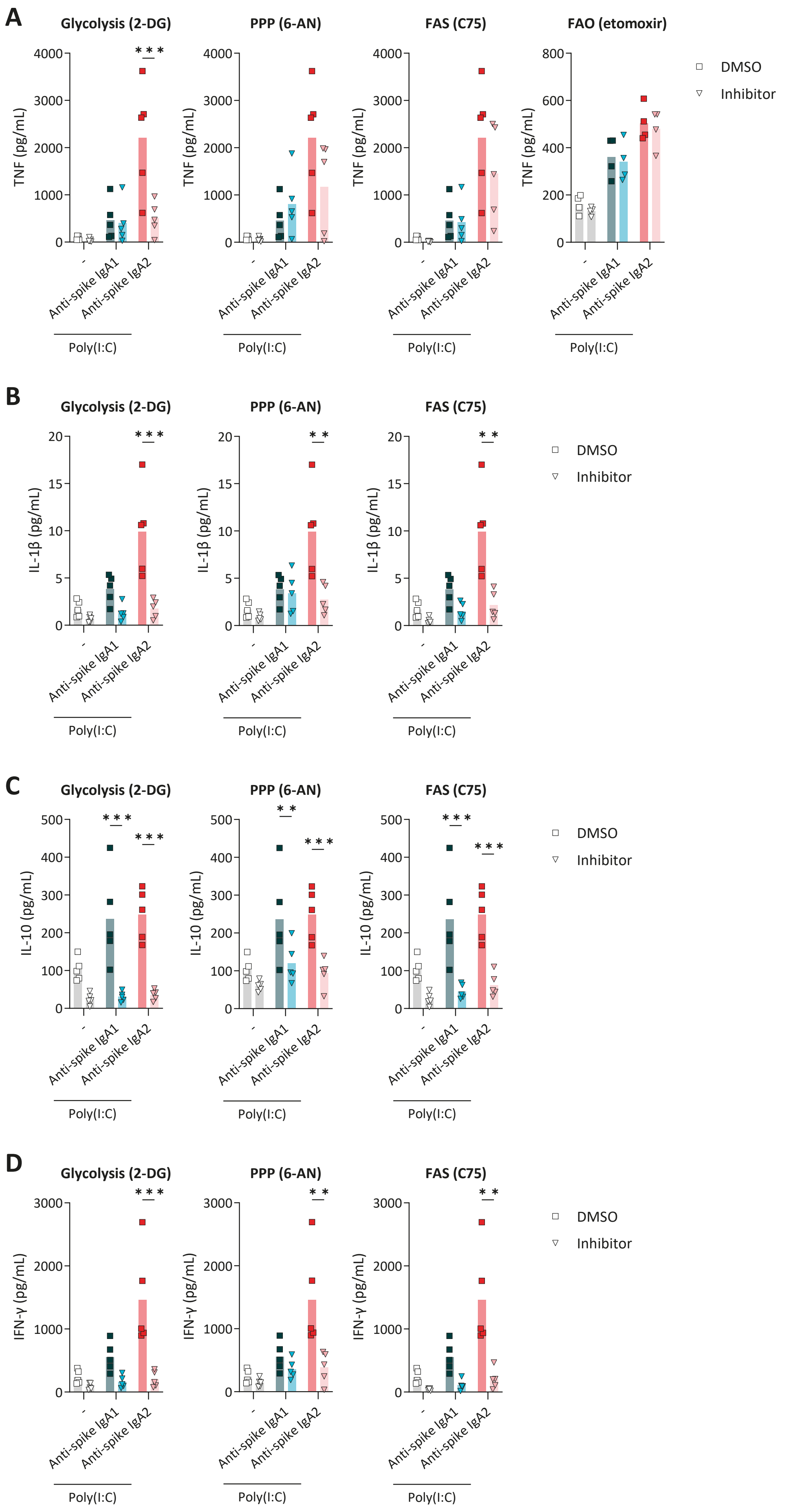

Figure S4. IgA2-induced metabolic reprogramming is reflected by a broad inflammatory cytokine profile. Before stimulation with Poly(I:C) and anti-spike IgA1 or -IgA2, macrophages were incubated with metabolic inhibitors targeting glycolysis, pentose phosphate pathway, fatty acid synthesis, or fatty acid oxidation. In addition to IL-6 (Fig. 3), samples were analyzed for TNF (A), IL-1β (B), IL-10 (C) and IFN-γ (D). Each dot represents a donor (n=4-6), statistical analysis by two-way ANOVA, *P<0.05, **P<0.01, ***P<0.001. PPP – pentose phosphate pathway; FAS – fatty acid synthesis; FAO – fatty acid oxidation.

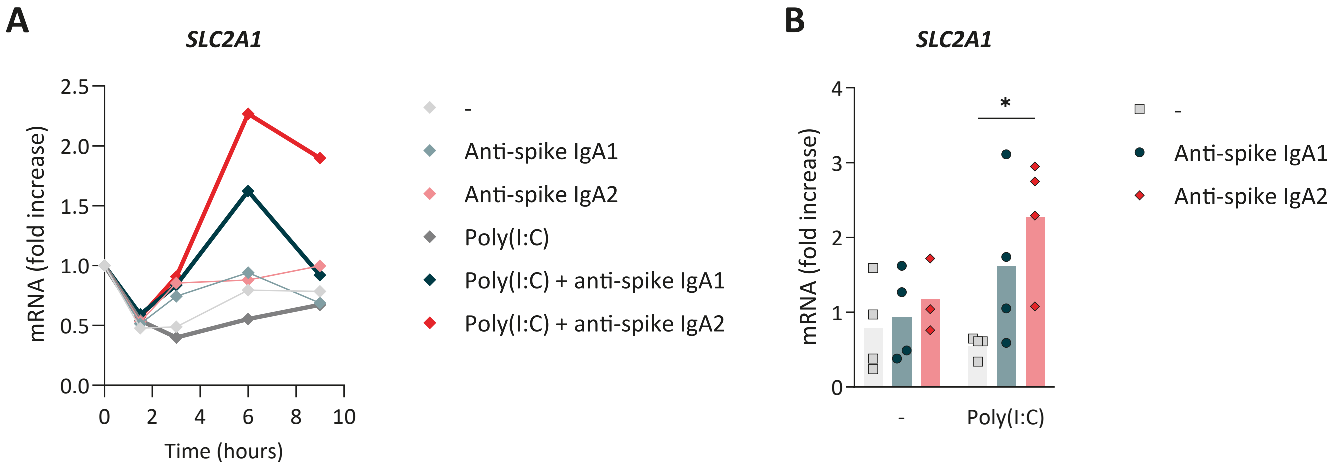

Figure S5. Gene expression of glucose transporter 1 GLUT1 was significantly induced by IgA2. mRNA expression of SLC2A1 was measured over time by qPCR, normalized to housekeeping genes, and presented as fold increase over T=0h (n=4) (A). The fold increase at T=6h is depicted in (B). Each dot represents a donor, with mean values (bar) per condition, analyzed by two-way ANOVA, *P<0.05.

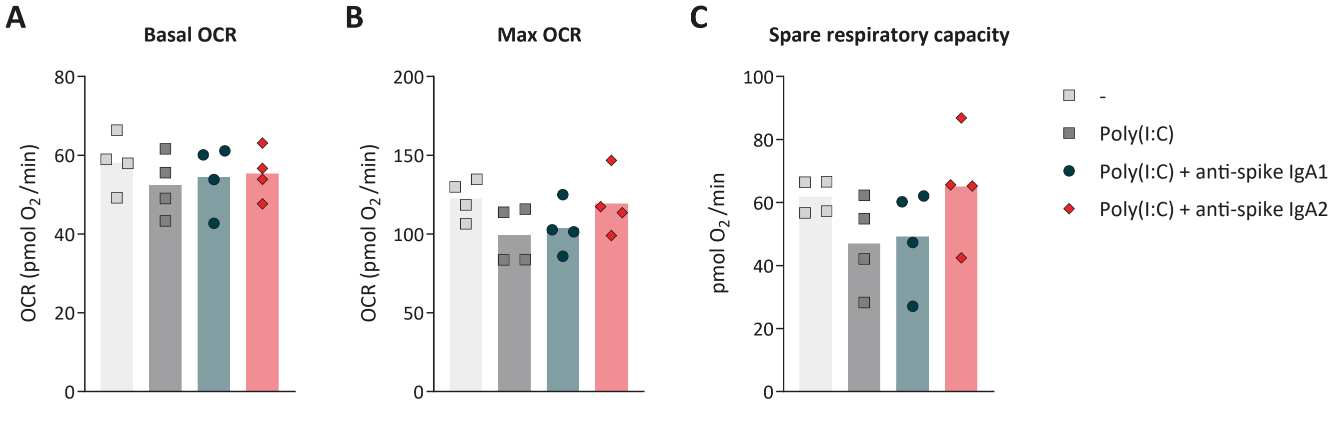

Figure S6. Stimulation of macrophages with IgA did not affect basal-, maximal-, and reserved oxygen consumption rates. Stimulated macrophages were analyzed for their extracellular flux. The oxygen consumption rate (OCR) and proton efflux rate (PER) were measured over time (Fig. 4) and basal- (A) and max (B) OCR, along with the spare respiratory capacity (C) were calculated. Data of 4 donors (dots) and mean values (bars).

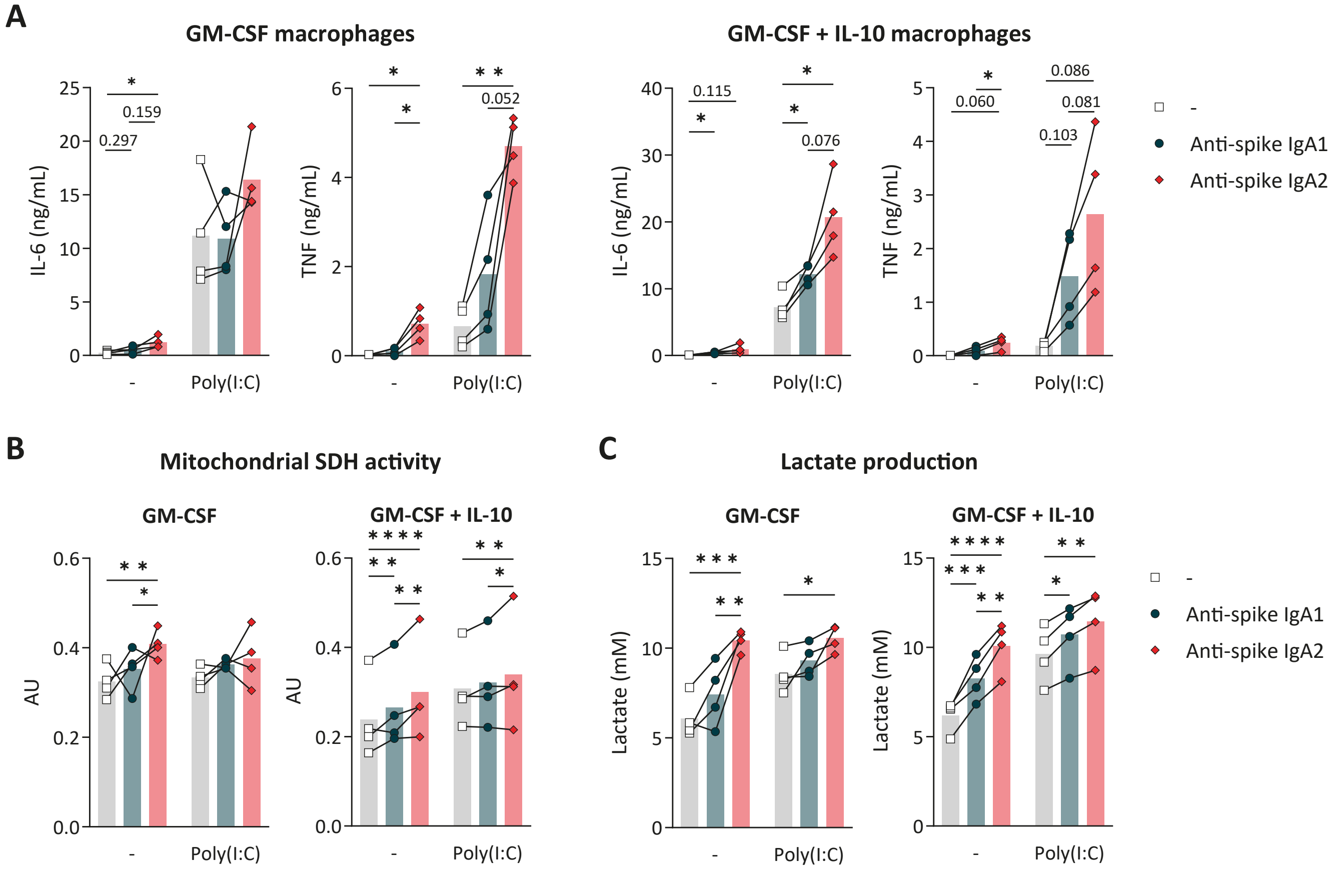

Figure S7. Cytokine and metabolic responses to IgA subclasses in GM-CSF-differentiated macrophages. Macrophages differentiated in the presence of GM-CSF with or without IL-10 stimulation were stimulated with Poly(I:C) and anti-spike IgA1 or -IgA2. IL-6 and TNF production (A), Mitochondrial SDH activity (B) and Lactate production (C) were measured by ELISA, MTT, and Lactate assays, respectively. Each dot or line represents a donor (n=4) with mean values (bars) per condition, statistical analysis by two-way ANOVA, *P<0.05, **P<0.01, ***P<0.001, **** p<0.0001.
